# Special Issue “Application Progress of Liposomes in Drug Development”

**DOI:** 10.3390/ijms25063454

**Published:** 2024-03-19

**Authors:** Carla M. Matos

**Affiliations:** FP-I3ID (Instituto de Investigação, Inovação e Desenvolvimento Fernando Pessoa), FP-BHS (Biomedical and Health Sciences), Universidade Fernando Pessoa, Praça 9 de Abril, 349, 4249-004 Porto, Portugal; cmatos@ufp.edu.pt

The second edition of the Special Issue entitled the “Application Progress of Liposomes in Drug Development” featured contributions predominantly focused on leveraging liposomes as enhancers and carriers in drug delivery in the context of cancer treatment, although this was not the initial intent of this Special Issue. This unintended thematic concentration highlights the persistent and significant interest in exploiting liposomal technologies specifically for cancer therapeutics within the scientific community.

Cancer is not a singular disease but rather a diverse collection of ailments, each characterized by distinct mechanisms of onset, affecting various organs, and yielding diverse outcomes. Cancer has persistently plagued humanity throughout its history, representing an enduring health challenge. The ongoing battle against cancer has evolved over time. Traditional cancer treatments, such as chemotherapy, often lack specificity, leading to severe side effects and suboptimal therapeutic outcomes [1].

Liposomes, a group of nanosized lipid-based carriers, have surfaced as highly promising entities for drug delivery in cancer treatment, providing a robust avenue for improving drug delivery specificity to cancer cells while mitigating systemic toxicity [2]. Notably, the pioneering strides in commercial liposome-based medicine development found their inception in the context of cancer treatment. The first liposome-containing medication commercially marketed was Doxil, which is a liposomal formulation of doxorubicin. Doxil was approved by the United States Food and Drug Administration (FDA) in 1995 for the treatment of certain types of cancer [3]. The liposomal encapsulation of doxorubicin allowed for a more controlled drug release, reducing systemic toxicity and improving treatment efficacy. Since then, other liposomal formulations have been developed for various therapeutic agents, and liposomal drug delivery remains an active area of research [4].

Liposomes can enhance the pharmacokinetics and biodistribution of drugs [5]. The encapsulation of drugs within liposomes protects them from degradation, increases their circulation time, and allows for preferential accumulation in tumor tissues through the enhanced permeability and retention (EPR) effect [2]. This selective targeting improves the therapeutic index and reduces off-target effects. These features are highlighted in the papers published by Moya-Garcia et al., Poudel et al., and Alrbyawi et al. [6,7,8].

Moya-Garcia et al. [6] explore the development and characterization of chitosan-coated liposomes (chitosomes) as a drug delivery system for docetaxel targeting laryngeal squamous cell carcinoma (LSCC). Their study is focused on evaluating the physical properties of the nanocarriers, including size, charge, stability, and mucoadhesiveness, as well as assessing their cytotoxic effects on cancer cells and healthy stromal cells. Docetaxel-loaded chitosomes exhibited higher toxicity toward LSCC compared to docetaxel-loaded liposomes, suggesting a potential therapeutic advantage of the chitosome formulation. They also investigated the drug release profile of docetaxel-loaded chitosomes, revealing a slower release compared to non-coated liposomes. The chitosomes demonstrated stability over a 35-day period, with a decrease in zeta potential values possibly attributed to the partial degradation of the chitosan coating. Their research presents a comprehensive characterization of the developed nanocarriers and provides promising in vitro results regarding their potential for targeted drug delivery to LSCC. Chitosan coatings appear to enhance the mucoadhesiveness and cytotoxicity of liposomes, which could be advantageous for localized drug delivery in the treatment of laryngeal cancer.

Poudel et al. [7] describe the preparation and characterization of a liposomal formulation of hispolon, a natural compound with potential anticancer properties. Their study involves the complexation of hispolon with sulfobutylether-β-cyclodextrin and its incorporation into liposomes. Their comprehensive solid-state characterization using techniques such as DSC, FTIR, NMR, and SEM provides strong evidence for the successful formation of stable inclusion complexes. The formulation development involving liposomes shows promise in achieving optimal characteristics, controlled drug release, and improved cytotoxicity against melanoma cell lines.

In another contribution, Alrbyawi et al. [8] describe the preparation and characterization of liposomal formulations for the delivery of the chemotherapeutic drug daunorubicin in breast cancer cells. Their cytotoxicity studies, particularly the determination of IC50 values and the observed enhanced cytotoxic effect of the liposomal formulation with cardiolipin compared to formulations without cardiolipin and free daunorubicin, support the potential effectiveness of the developed liposomes in inhibiting cancer cell growth.

Photodynamic therapy is a local cancer treatment that uses the therapeutic potential of light for treatment. This technique involves the use of light to activate a photosensitizer, which then releases reactive oxygen species to destroy the irradiated tissue. Photodynamic therapy has the potential to enhance drug delivery by altering the permeability of the tumor vasculature [9]. The encapsulation of photosensitizers within liposomes further augments their accumulation within the tumor, enhancing the efficacy and precision of the treatment, especially when used in combination with other anticancer drugs [10].

The study by Lazaro-Carrillo et al. [11] presents an innovative approach by coencapsulating the chemotherapeutic drug irinotecan and the photosensitizer protoporphyrin IX within liposomes for potential chemophototherapy. Through confocal microscopy, the colocalization of the two drugs in the cytoplasm and their partial colocalization at the plasma membrane were observed, suggesting potential synergistic interactions. The authors report the successful formulation of bimodal liposomes and systematically evaluate their physicochemical characteristics, stability, and cytotoxicity against HeLa cells. Their study contributes to the growing field of nanomedicine and combination therapies, showcasing the potential of liposomal formulations for the codelivery of chemotherapeutic agents and photosensitizers. Their comprehensive evaluation of physicochemical characteristics, stability, and long-term cellular responses strengthens the significance of their findings.

Liposomes have been employed as artificial cells, serving as valuable tools for investigating biological interactions occurring at cell membranes. This includes studying phenomena like viral infections or unraveling the mechanistic intricacies of cancer. By mimicking certain aspects of cell membranes, liposomes provide researchers with a controllable and customizable platform to gain insights into complex cellular processes, facilitating a deeper understanding of the molecular interactions involved in various biological phenomena [12].

In this context, Nguyen et al. [13] outline the preparation and characterization of cysteine-encapsulated liposomes for investigating molecular interactions at the membrane. The liposomes were designed to release encapsulated cysteine upon membrane disruption, inducing the aggregation of gold nanoparticles and resulting in color change. Phospholipase A2 was used as a model enzyme to demonstrate the system’s applicability to investigate enzymatic activities. The phospholipase A2-induced hydrolysis of lipids resulted in the release of cysteine from liposomes, leading to the aggregation of gold nanoparticles and color change. The system was successfully utilized to analyze the enzymatic activity occurring at the membrane interface.

Liposomes have emerged as versatile and effective drug delivery vehicles for cancer treatment, offering improved pharmacokinetics, targeted delivery, and potential solutions to overcome drug resistance. While challenges remain, ongoing research and clinical trials suggest that liposomal formulations hold great promise in shaping the future of personalized and targeted cancer therapy. As technology advances and our understanding of cancer biology deepens, liposomes play a crucial role in the next generation of anticancer drug development.
